# ^177^Lu-PSMA (R)Evolution in Cancer Care: Is It Really Happening?

**DOI:** 10.2967/jnumed.124.267951

**Published:** 2024-09

**Authors:** Andreas Poschenrieder, Jasminka Taleska, Leonhard Schaetz

**Affiliations:** 1 Advanced Accelerator Applications, Novartis, Geneva, Switzerland; and; 2 Novartis Pharma AG, Basel, Switzerland

Radiopharmaceutical therapy (RPT) is established as an innovative, effective, and well-tolerated treatment platform approved for well-differentiated neuroendocrine tumors and advanced prostate cancer (PC) and is being investigated in many other cancers such as breast, lung, and thyroid (1–3), emerging as an essential pillar in oncology.

On the basis of several seminal clinical studies, [^177^Lu]Lu-prostate-specific membrane antigen-617 (hereafter referred to as ^177^Lu-PSMA-617) is now integrated into international clinical guidelines for PC and is gaining significant traction among patients and physicians alike because of its proven efficacy, favorable safety profile, and ability to maintain quality of life longer than other therapies, for example, chemotherapy (3–5). Efforts to expand capacities for manufacturing ^177^Lu-PSMA-617 have successfully addressed postapproval supply shortages encountered in the United States and Europe, leading to the projected availability of 250,000 RPT doses in 2024 (6). Although this shows that RPT can be produced at industrial scale to meet increasing demand, the readiness of health care systems (HCSs)—in other words, the availability of health care professionals, policies, processes, infrastructure, and resources to deliver RPT in a timely and equitable manner—remains highly variable. With a projected exponential growth in RPT (7*,*^8^) as many pharmaceutical companies invest in theranostics, now is the right time to ask: Is a ^177^Lu-PSMA (r)evolution in cancer care really happening?

In this article, we explore the opportunities and challenges in integrating RPT into everyday clinical practice and the changes needed by HCSs to establish this therapy as an essential pillar in oncology care.

## A ^177^Lu-PSMA REVOLUTION IN ONCOLOGY: THE TIPPING POINT

### The Clinical Value of RPT

RPT has gained considerable momentum in recent years, with a series of pivotal trials (e.g., VISION (3), PSMAfore (9), TheraP (5), and SPLASH (10)) demonstrating the benefits of ^177^Lu-PSMA–based RPTs, including reduced risks for radiographic disease progression and death, against standards of care in patients with previously treated, advanced PC. Many more RPTs are under investigation across the PC treatment landscape.

### Unveiling RPT Future Potential: Mergers and Acquisitions

Since PSMA can also be expressed in other cancers, ^177^Lu-PSMA–based RPT holds the promise of expanding into new indications (2). The global theranostics market is forecasted to grow exponentially from $7.2 billion in 2022 to $39 billion by 2032, indicating the potential transformative value of RPT in oncology (7). After Novartis’ acquisition of 2 pioneering RPT companies, Endocyte (11) and Advanced Accelerator Applications (12), in 2018 and subsequent successful clinical trials, other world-leading pharmaceutical companies are following suit: the acquisition of POINT Biopharma, RayzeBio, and Fusion Pharmaceuticals by Eli Lilly, Bristol Myers Squibb, and AstraZeneca, respectively (13). Novartis further strengthened this commitment with 2 recent deals, agreeing to acquire Mariana Oncology (14) and expanding a collaboration with PeptiDream (15).

Given this uptick in radiopharmaceutical acquisitions, the anticipated increasing number of clinical trials and product approvals, the rising number of cyclotrons operated in nuclear medicine across central Europe (16), and the estimated number of centers needed across the United States (17), it is clear that ^177^Lu-PSMA–based RPT has established a foothold in oncology care. The application and importance of PSMA PET/CT are expanding. As nuclear medicine evolves to offer theranostic services including RPT, it becomes an increasingly important aspect for multidisciplinary team (MDT) stakeholders to consider in PC management. Nuclear medicine integration into MDT practice and referral pathways can enhance its role in oncology care to benefit patient lives. However, these novel treatments come with challenges that need to be addressed.

## SCALING RPT TO WIDER CLINICAL PRACTICE: THE CHALLENGES

Bringing novel treatments to real-world clinical practice requires more than just positive clinical trials and regulatory approval. Aspects of treatment reimbursement are also important, though not discussed in this article since this varies widely among countries, indications, and clinical benefits of current and future approved RPTs. The increasingly urgent need to integrate transformative treatments into the PC standard of care cannot be overstated. To optimize patient outcomes and maintain a competitive edge, HCSs worldwide should keep pace with innovative treatment modalities, adapt quickly to ensure readiness, and facilitate straightforward and equitable access. Failure to do so poses the risk of constraining patients to suboptimal treatment options, resulting in diminished survival and compromised quality of life. The main priorities to ensure HCSs’ readiness for RPT consist of awareness and education; appropriate infrastructure, including patient referral and management systems; and harmonization of regulatory frameworks.

### The Need for Awareness and Education

Future expansion of RPT indications and trials will lead to intensification of demand, increasing the strain on HCSs, with many centers already running at full capacity. RPTs were historically available only in highly specialized or academic centers designed to treat a relatively small number of patients. To meet the surge in ^177^Lu-PSMA and theranostics demand, capacity needs to be increased substantially and supported by reregulation. Given the global heterogeneity in discharging patients after RPT administration and its impact on capacity, regulations should be harmonized and favor evidence-based, patient-centric release criteria. A dedicated workforce comprising, but not limited to, urologists, medical oncologists, nuclear medicine physicians, and radiation oncologists collaborating within MDTs is critical to achieving this expansion. Therefore, engaging and educating referring physicians, and involving the whole MDT as part of the decision-making process, are important. Attracting and training talented patient-facing staff to manage patients’ ongoing needs and expectations are also crucial. Ultimately, efficient delivery and patient-centric release criteria are critical for broader RPT use, especially for centers that have yet to set up nuclear medicine services.

### Referrals and the MDT

Referral of eligible patients and the role of the MDT are crucial for enabling access to innovative medicines. The MDT in PC should encompass a broad range of specialties, all collaborating closely to choose the optimal treatment for each patient. PSMA PET radioligand imaging agents—for example, ^68^Ga-gozetotide, ^18^F-piflufolastat, and ^18^F-flotufolastat—are also increasingly used for diagnosis or treatment selection (13). Alongside RPT, such radioligand imaging agents offer an innovative informed approach to targeted treatment decisions, making the nuclear medicine physician an important member of the MDT.

### Investment in Infrastructure and Optimization of Existing Processes

Given the unique properties of radiopharmaceuticals, hospitals require appropriate infrastructure, including lead shielding, storage, handling and disposal systems, and specialized staff to safely administer treatments. Advances in nuclear medicine PC treatment have naturally led to increased HCS investments to improve and modernize existing infrastructure, thereby paving the way for the integration of RPT in wider cancer care (18). For instance, treatment centers are being upgraded with PET/CT or SPECT/CT scanners and radiopharmaceutical handling facilities to accommodate safe and efficient administration of RPTs. From a practical perspective, the time from diagnosis to ^177^Lu-PSMA treatment initiation reflects the speed of the PSMA PET imaging, diagnosis, and referral processes. This period varies significantly among geographies and is currently estimated at 2 wk in the United States, 3 wk in France, 7 wk in Spain, and up to 8 wk in Germany (19*,*^20^). Because metastatic castration-resistant PC is a rapidly progressing disease, it is vital to minimize delays in imaging and treatment initiation. Furthermore, the number of identified centers with adequate radiation-handling facilities, up-to-date PET/CT and SPECT scanners, and protective equipment for health care professionals is limited. In short, infrastructure is often tailored to patient numbers of the past, when radiation medicine was focused on imaging rather than treatment; the current number of RPT centers is estimated at 30–35 in Italy (population, ∼60 million), 40–45 in both Germany (population, ∼83 million) and France (population, ∼68 million), 65–70 in Spain (population, ∼50 million), and over 200 in the United States (population, ∼330 million) (19*,*^20^). The current capacity of HCSs to diagnose and treat patients with radiopharmaceuticals should be assessed carefully to understand infrastructure barriers and optimize the current model to meet anticipated future demands.

Lack of funding for theranostics can also hinder equitable access because of varying levels of insurance coverage, compounded, in the case of many European countries, by restricted reimbursement to certain patient populations.

### Development of Harmonized Regulatory Frameworks

RPT development, licensing, and delivery fall within the remit of both radioprotection and pharmaceutical regulatory frameworks. Both frameworks date from a time when therapeutic radiopharmaceuticals were niche products and randomized industry-led registration trials and product approvals were the exception. Therefore, no differentiation between licensed and unlicensed products is being made in the context of Council Directive 2013/59/Euratom, the Basic Safety Standards Directive, a legal framework established to ensure the highest level of safety for medical ionizing radiation procedures (21). Furthermore, specific guidelines for the development and licensing of innovative radiopharmaceuticals such as RPTs and radioligand imaging are missing in pharmaceutical legislation. Within Europe, there is significant heterogeneity within local regulatory frameworks for radiopharmaceuticals because of different interpretations of what is presently written in the European Directives (21*,*^22^). There is a need for harmonized, simple, and adequate regulations facilitating patient access to RPTs. An example of this is the need to hospitalize patients after ^177^Lu-PSMA treatment, which varies both regionally and globally: hospitalization is not strictly necessary in the United States (23), France, and Spain; patients may be released the same day in some regions of Italy; whereas in Germany, patients are required to stay in the hospital for at least 48 h, impacting capacity, accessibility, and the patient (19). Because radiopharmaceuticals are moving from niche to broad adoption in oncology, there is a need for adequately adapted regulatory frameworks. These need to provide better clarity on the roles of the different agencies governing these products, so that the dual oversight is seamlessly integrated and no longer hampers the conduct of clinical trials that involve innovative RPTs and radioligand imaging by making conflicting demands or requiring access to licensed radiopharmaceuticals. In this regard, the currently ongoing update of European Union pharmaceutical legislation is an important opportunity—which must not be missed—to revise definitions and regulatory pathways for radiopharmaceuticals that account for major developments in the field.

## CONCLUSION AND FUTURE OUTLOOK

The field of theranostics holds enormous promise for the future of cancer management. The significant growth seen in the global theranostics market can be attributed to multiple factors, including the proven clinical value of approved RPT agents and their impact on patients’ lives, alongside their successful commercialization. The investigation of different therapeutic targets across multiple disease areas, the use of combination therapies, and the exploration of different nuclides are only some of the areas that could lead to further advancements. Several challenges have been mentioned, including improving education and awareness, implementing successful referral systems, harmonizing regulations, and expanding infrastructure. To conclude, in the background of continued strategic investment due to the striking potential for patients, the ^177^Lu-PSMA (r)evolution in cancer care must happen and will happen, but key stakeholders must closely collaborate to ensure that HCSs can allow timely and equitable access to treatment.

## DISCLOSURE

All authors are employees of Novartis and are Novartis shareholders. No other potential conflict of interest relevant to this article was reported.
